# Supplementary material for: Impact of Rehabilitation on Physical and Neuropsychological Health of Patients Who Acquired COVID-19 in the Workplace
Source: Int J Environ Res Public Health. 2023 Jan 13;20(2):1468. doi: 10.3390/ijerph20021468 (PMC9864141; doi:10.3390/ijerph20021468)
Supplement: Supplementary file 1 [file ijerph-20-01468-s001.zip › ijerph-2136482-supplementary.pdf]

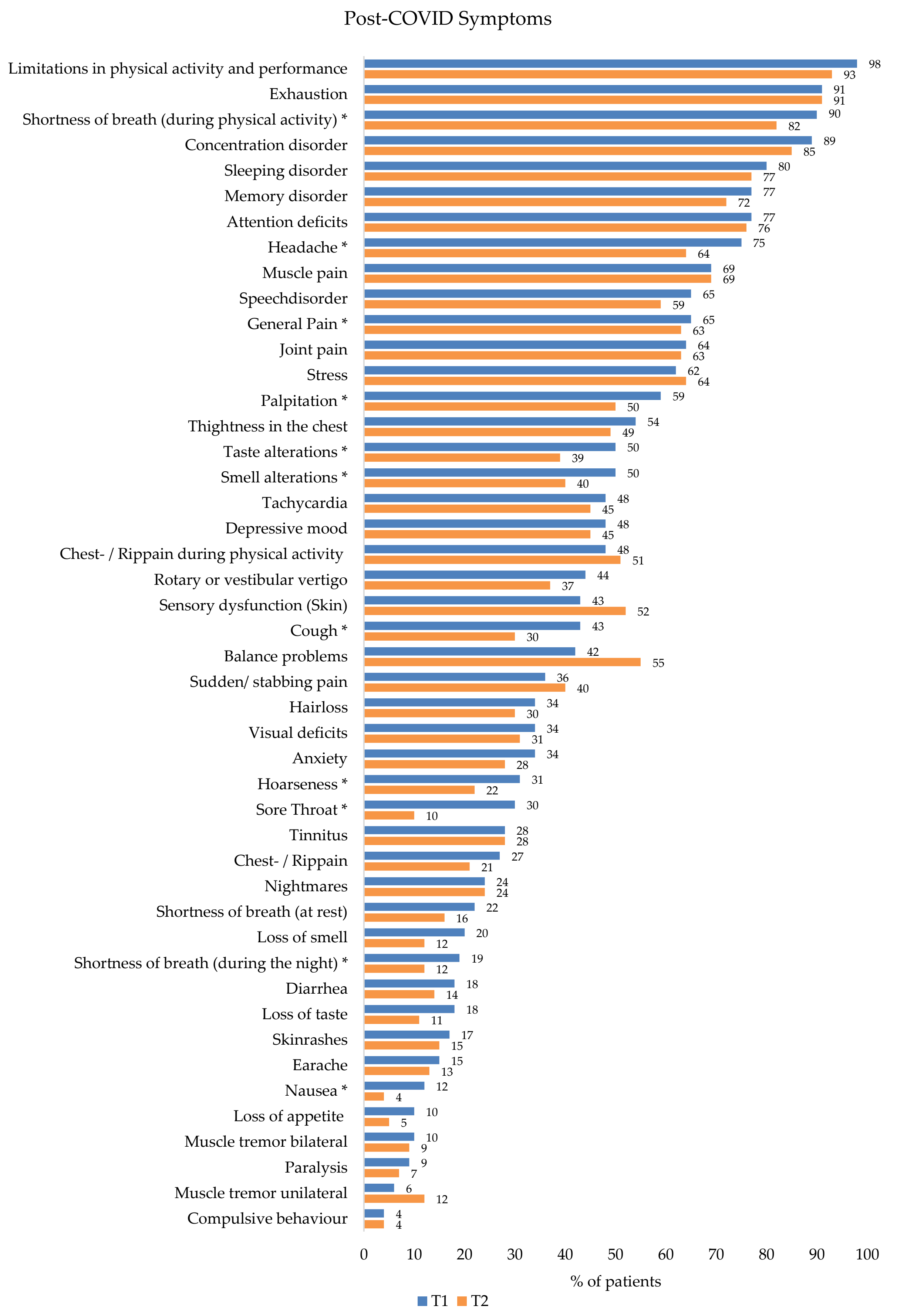

Figure S1: Ongoing symptoms related to long-/post-COVID before (T1. blue) and after (T2. orange) rehabilitation. Significant differences between T1 and T2 are marked with * ($p < 0.05$).

Table S1: Groupwise comparison of patients with a mild/moderate COVID-19 infection and a severe/critical COVID-19 infection at timepoint T1

	Mild/Moderate COVID-19		Severe/ critical COVID-19		Between-group differences		
	**N**	**Median (IQR)**	**N**	**Median (IQR)**	**z**	**p**	**r**
Physical Performance							
6MWD [m]	91	525.00 (440.00-583.00)	36	498.50 (447.75-542.75)	-1.388	0.165	-0.123
1MSTST	90	20 (16-24)	36	17.50 (14-22)	-1.568	0.117	-0.140
Handgrip strength [kg]	91	27.90 (20.83-35.30)	36	26.90 (20.33-34.83)	0.027	0.979	0.002
Quadriceps strength [kg]	91	92.96 (69.60-130.38)	36	103.59 (65.91-133.74)	-0.045	0.964	-0.004
Subjective physical ability	90	4.78 (3.53-6.03)	36	4.61 (3.03-7.17)	-0.829	0.407	-0.074
Neuropsychological Parameters							
MoCA score	91	27 (26-29)	36	26.5 (25-28)	-2.598	0.009	-0.231
DSST	91	47 (39-54)	36	41.5 (34-51.5)	-2.283	0.022	-0.203
HADSdepression	91	7 (4-10)	36	6 (3-9.3)	1.210	0.226	0.108
HADSanxiety	91	6 (4-10)	36	5 (3-10)	1.151	0.250	0.102
FIS	91	89 (74-113)	36	93.5 (63.8-113)	0.701	0.483	0.063
BFI	91	5.6 (4.7-6.6)	36	5.3 (4.6-6.6)	0.187	0.851	0.017
Subjective mental health	91	5.4 (4.5-6.7)	36	5.5 (4.9-7.1)	-0.286	0.775	-0.025
Work ability							
WAI score	88	25 (21-28)	35	24 (20-28)	-0.559	0.576	-0.050
• Work ability	90	3 (0.75-5)	36	2 (0-4.75)	-0.882	0.378	-0.079
• Work requirements	89	7 (6-8.75)	36	7.75 (6-9)	0.705	0.481	0.063

IQR = interquartile range.

Table S2: Groupwise comparison of male and female patients at timepoint T1

	Male		Female		Between-group differences		
	**N**	**Median (IQR)**	**N**	**Median (IQR)**	**z**	**p**	**r**
Physical Performance							
6MWD [m]	30	532 (463.00-588.25)	97	517 (432.00-557.50)	-1.084	0.278	-0.096
1MSTST	30	18.50 (14.75-23.25)	96	20 (16-24)	0.938	0.348	0.084
Handgrip strength [kg]	30	38.35 (32.38-49.12)	97	25.43 (20.08-31.32)	-5.480	< 0.001	-0.486
Quadriceps strength [kg]	30	134.99 (115.54-145.81)	97	85.19 (65.23-113.05)	-4.975	< 0.001	-0.442
Subjective physical ability	30	5.72 (3.94-7.00)	96	4.61 (3.14-5.44)	-2.559	0.011	-0.228
Neuropsychological Parameters							
MoCA score	30	27 (25-28.3)	97	27 (26-28)	0.301	0.763	0.027
DSST	30	41 (34.5-47.5)	97	47 (38.5-54)	2.604	0.009	0.231
HADSdepression	30	8 (3.8-10.3)	97	7 (4-11)	0.287	0.774	0.023
HADSanxiety	30	5 (3-10.3)	97	7 (4-11)	1.022	0.307	0.091

FIS	30	83.5 (60.3-110.5)	97	99 (77.5-115)	2.186	0.029	0.194
BFI	30	5.2 (3.9-6.7)	97	5.6 (4.7-7)	1.411	0.158	0.125
Subjective mental health	30	5.6 (4.6-7.0)	97	5.3 (4.5-6.3)	-1.391	0.164	-0.123
Work ability							
WAI score	28	24.25 (20.63-29.5)	95	25 (21-28)	-0.269	0.788	-0.024
• Work ability	30	4 (1.75-5.25)	96	3 (0-5)	-1.133	0.257	-0.101
• Work requirements	30	7 (6-9)	95	7.5 (6-9)	0.800	0.424	0.072

IQR = interquartile range.

Table S3: Groupwise comparison of healthcare- and non-healthcare workers at timepoint T1

	Healthcare Workers		Non-Healthcare Workers		Between-group differences		
	N	Median (IQR)	N	Median (IQR)	z	p	r
Physical Performance							
6MWD [m]	89	517.00 (436.50-557.50)	38	537.50 (461.75-583.25)	1.227	0.220	0.109
1MSTST	88	20 (16-23.75)	38	20.50 (14.50-25)	0.090	0.928	0.008
Handgrip strength [kg]	89	26.87 (20.22-35.03)	38	31.35 (24.00-36.08)	1.264	0.206	0.112
Quadriceps strength [kg]	89	88.82 (67.10-120.97)	38	124.16 (83.62-141.38)	2.788	0.005	0.247
Subjective physical ability	88	4.78 (3.44-6.08)	38	4.44 (3.28-6.22)	-0.787	0.431	-0.070
Neuropsychological Parameters							
MoCA score	89	27 (26-28)	38	26.5 (25-28)	-0.671	0.502	-0.056
DSST	89	47 (39.5-53)	38	41.5 (34-51.5)	-1.475	0.140	-0.131
HADSdepression	89	8 (5-11)	38	6 (3-9.3)	-1.544	0.123	-0.137
HADSanxiety	89	7 (4-11)	38	5 (3-10)	-1.748	0.080	-0.155
FIS	89	98 (74.5-113.5)	38	93.5 (63.8-113)	-0.829	0.407	-0.074
BFI	89	5.7 (4.4-7.1)	38	5.3 (4.6-6.6)	-0.487	0.626	-0.043
Subjective mental health	89	5.3 (4.2-6.3)	38	5.5 (4.9-7.1)	1.764	0.078	0.157
Work ability							
WAI score	85	24 (20.75-27)	38	25.25 (21-31)	1.236	0.219	0.111
• Work ability	88	3 (0-5)	38	3.5 (0.75-5.25)	0.943	0.345	0.084
• Work requirements	87	7.5 (6-9)	38	7 (6-8.63)	-1.163	0.245	-0.104

IQR = interquartile range.

Table S4: Groupwise comparison of mild/moderate and severe/critical COVID-19 patients regarding the outcomes of an inpatient rehabilitation program

	Mild/ moderate COVID-19				Severe/ critical COVID-19				Between-group difference		
	N	Pre Median (IQR)	Post Median (IQR)	Δ	N	Pre Median (IQR)	Post Median (IQR)	Δ	z	p	r
Physical Performance											

	N	Pre Median (IQR)	Post Median (IQR)	Δ	N	Pre Median (IQR)	Post Median (IQR)	Δ	z	p	r
6MWD [m]	87	525.00 (440.00-583.00)	584.00 (522.00-644.00)	59.00 (20.00-112.00)	32	498.50 (447.75-542.75)	571.50 (522.50-611.75)	78.50 (44.00-122.75)	1.013	0.311	0.093
1MSTST	86	20 (16-24)	22 (18-26)	2 (-1-5)	32	17.50 (14-22)	20 (16.25-24.50)	2 (0.25-4)	0.407	0.684	0.038
Handgrip strength [kg]	89	27.90 (20.83-35.30)	30.37 (20.98-36.58)	-0.13 (-2.32-4.75)	32	26.90 (20.33-34.83)	28.68 (21.38-33.49)	-0.50 (-3.26-3.45)	-1.061	0.289	-0.097
Quadriceps strength [kg]	88	92.96 (69.59-130.37)	110.13 (89.83-146.73)	12.75 (0.54-25.57)	32	103.59 (65.9-133.74)	114.39 (81.06-144.57)	13.82 (-0.24-34.14)	-0.199	0.842	-0.018
Subjective physical ability	79	4.78 (3.56-6)	6.00 (4.67-7.22)	1.22 (0.22-2.22)	33	4.61 (3.03-6.19)	5.33 (3.94-7.17)	0.56 (0.11-2.44)	-0.537	0.592	-0.051
Neuropsychological Parameters											
MoCA	88	27 (26-29)	27.5 (26-29)	0 (-1-2)	34	26 (25-27)	27 (25.8-28.3)	1 (-1-2)	0.966	0.334	0.088
DSST	88	47 (39-54)	51 (42-58)	2 (-1-6)	34	41 (34.3-49.8)	46.5 (35.8-54.5)	4 (-0.25-6.3)	0.701	0.483	0.064
HADSdepression	88	7 (4-10)	5 (3-9)	-1 (-3-0)	34	9 (4.3-11.8)	7 (3-11)	-1 (-3-0.25)	-0.280	0.780	-0.025
HADSanxiety	88	6 (4-10)	5 (2.5-9)	-1 (-3-0)	34	7.5 (4-12)	7 (3-11)	-1.5 (-2-0)	-0.211	0.833	-0.019
FIS	87	89 (74-113)	82 (65-111)	-4 (-16-4)	33	101.5 (69.3-113.5)	92 (49.5-115)	-7 (-22-5)	-0.703	0.482	-0.064
BFI	88	5.6 (4.7-6.6)	5.2 (3.8-6.3)	-0.33 (-1-0.33)	34	5.5 (4-7.3)	5.6 (3.8-7.1)	-0.06 (-1.2-0.69)	0.911	0.362	0.082
Subjective mental health	88	5.4 (4.5-6.6)	6.0 (4.5-7.2)	0.27 (-0.43-1.27)	34	5.2 (4.5-6.3)	5.8 (4-7.7)	0.5 (-0.4-1.3)	0.306	0.760	0.027
Work Ability											
WAI score	82	25 (21-28)	24.25 (22-28)	0 (-2-2)	33	24 (20-28)	25.25 (20-31)	1 (-1-2)	1.444	0.149	0.134
• Work ability	84	3 (1-5)	3 (1-5.75)	0 (-1-1.75)	34	2 (0-4.75)	3 (0-6)	0 (0-2)	1.271	0.204	0.117
• Work requirements	83	7 (6-8.63)	7 (6-8)	0 (-1-1)	34	7.75 (6-9)	8 (6-9.13)	0 (-1-0.25)	0.607	0.544	0.056

IQR = interquartile range.

Table S5: Groupwise comparison of male and female COVID-19 patients regarding the outcomes of an inpatient rehabilitation program

	Male				Female				Between-group difference		
	**N**	**Pre Median (IQR)**	**Post Median (IQR)**	**Δ**	**N**	**Pre Median (IQR)**	**Post Median (IQR)**	**Δ**	**z**	**p**	**r**
Physical Performance											
6MWD [m]	28	532.00 (463.00-588.25)	592.50 (534.25-687.00)	77.00 (45.50-102.25)	91	517.00 (432.00-557.50)	576.00 (520.00-627.00)	59.00 (25.00-122.00)	-0.044	0.965	-0.004
1MSTST	29	18.50 (14.75-23.25)	19 (16-24.50)	1 (-1-3)	89	20 (16-24)	22 (18-27)	2 (0-5.50)	1.740	0.082	0.160
Handgrip strength [kg]	29	38.35 (32.38-49.12)	39.33 (30.18-48.45)	-0.53 (-5.85-5.88)	92	25.43 (20.08-31.32)	26.87 (19.90-32.15)	-0.15 (-2.15-3.28)	0.452	0.651	0.041
Quadriceps strength [kg]	29	134.98 (115.53-145.80)	146.81 (125.16-182.35)	10.91 (0.14-31.79)	91	85.18 (65.23-113.04)	103.70 (84.18-126.22)	12.94 (0.60-30.33)	0.285	0.776	0.026
Subjective physical ability	25	5.72 (3.94-7)	6.56 (4-7.83)	1.00 (-0.61-1.78)	87	4.56 (3.17-5.44)	5.78 (4.56-7.11)	1.22 (0.22-2.44)	1.661	0.097	0.157
Neuropsychological Parameters											
MoCA	29	27 (25-28.3)	27 (27-28)	0 (-1-1.5)	93	27 (26-28)	27 (26-29)	0 (-1-2)	0.576	0.565	0.052

	N	Pre	Post	Δ	N	Pre	Post	Δ	z	p	r
DSST	29	41 (34.5-47.5)	43 (35-51.5)	2 (-2.5-5)	93	47 (38.5-54)	51 (43-57.5)	3 (-1-6.5)	0.428	0.428	0.039
HADSdepression	29	8 (3.8-10.3)	5 (2.5-10.5)	-1 (-1-0.5)	93	7 (4-11)	6 (3-9)	-1 (-3-0)	-0.513	0.608	-0.046
HADSanxiety	29	5 (3-10.3)	5 (2-9)	-1 (-2-0)	93	7 (4-11)	6 (3-10)	-1 (-3-0)	-0.505	0.614	-0.045
FIS	29	83.5 (60.3-110.5)	71 (49.5-112.5)	-2 (-12.5-4.5)	91	99 (77.5-115)	87 (68-111)	-6 (-21-4)	-0.782	0.434	-0.071
BFI	29	5.2 (3.9-6.7)	4.7 (3.1-7.3)	-0.22 (-0.94-0.39)	93	5.6 (4.7-7)	5.3 (4-6.4)	-0.22 (-1.1-0.47)	0.289	0.773	0.026
Subjective mental health	29	5.6 (4.6-7.0)	6.2 (4.2-7.9)	0.36 (-0.36-1.36)	93	5.3 (4.5-6.3)	5.8 (4.5-7)	-0.27 (-0.41-1.1)	0.078	0.938	0.007
Work Ability											
WAI score	25	24.25 (20.63-29.5)	25 (19.38-29.5)	0 (-2-1.5)	90	25 (21-27.75)	24.75 (21-28)	0 (-2-3)	0.490	0.624	0.045
• Work ability	27	4 (1.75-5.25)	4 (1-6)	0 (-1-2)	91	3 (0-5)	3 (0-6)	0 (-1-2)	0.160	0.987	0.014
• Work requirements	27	7 (6-9)	6 (5-8)	0 (-1-0)	90	7.5 (6-9)	7 (6-8)	0 (-1-1)	0.617	0.537	0.057

IQR = interquartile range.

Table S6: Groupwise comparison of healthcare and non-healthcare workers regarding the outcomes of an inpatient rehabilitation program

	**Healthcare workers**				**Non-healthcare workers**				**Between-group difference**		
	**N**	**Pre Median (IQR)**	**Post Median (IQR)**	**Δ**	**N**	**Pre Median (IQR)**	**Post Median (IQR)**	**Δ**	**z**	**p**	**r**
Physical Performance											
6MWD [m]	84	517.00 (436.50-557.50)	574.50 (520.50-626.00)	77.50 (32.00-120.75)	35	537.50 (461.75-583.25)	587 (539.00-663.00)	50.00 (25.00-85.00)	-1.286	0.198	-0.118
1MSTS	83	20 (16-23.75)	22 (18-26)	2 (-1-5)	35	20.50 (14.50-25)	22 (17-27)	2 (-1-4)	-0.396	0.692	-0.037
Handgrip strength [kg]	85	26.87 (20.22-35.03)	27.90 (21.02-34.27)	-0.17 (-2.42-4.77)	36	31.35 (24.00-36.08)	30.47 (21.03-37.16)	-0.15 (-4.38-2.98)	-0.544	0.586	-0.050
Quadriceps strength [kg]	84	88.81 (67.1-120.96)	105.61 (86.41-136.41)	13.02 (0.71-31.55)	36	124.16 (83.6-141.37)	124.69 (103-158.86)	12.47 (0.04-25.18)	-0.395	0.693	-0.036
Subjective physical ability	78	4.78 (3.44-6.06)	5.78 (4.53-7.22)	1.17 (0.08-2.22)	34	4.44 (3.28-6.22)	5.78 (4.53-7.17)	1.00 (0.22-2.25)	0.063	0.950	0.006
Neuropsychological Parameters											
MoCA	85	27 (26-28)	27 (26-29)	0 (-1-2)	37	26.5 (25-28)	27 (25-29)	0 (-1-2)	-0.595	0.552	-0.054
DSST	85	47 (39.5-53)	51 (43.5-57)	3 (-0.5-6)	37	41.5 (34-51.5)	44 (35-56)	0 (-3-6)	-1.554	0.120	-0.141
HADSdepression	85	8 (5-11)	6 (3-10)	-1 (-3-0)	37	6 (3-9.3)	5 (4-10.5)	0 (-1-1)	2.152	0.031	0.194
HADSanxiety	85	7 (4-11)	6 (3-10)	-2 (-3-0)	37	5 (3-10)	4 (2.5-9)	-1 (-2-0.5)	1.458	0.145	0.132
FIS	83	98 (74.5-113.5)	85 (65-111)	-6 (-22-6)	37	93.5 (63.8-113)	88 (64.5-113)	-3 (-11-3.5)	0.873	0.383	0.08
BFI	85	5.7 (4.4-7.1)	5.3 (3.6-6.7)	-0.33 (-1.3-0.44)	37	5.3 (4.6-6.6)	5.3 (3.9-6.4)	-0.11 (-0.72-0.33)	0.986	0.324	0.089
Subjective mental health	85	5.3 (4.2-6.3)	5.8 (4.5-7.3)	0.45 (-0.18-1.18)	37	5.5 (4.9-7.1)	6 (4.7-7.2)	0.9 (-0.59-1.4)	-1.315	0.188	-0.119
Work Ability											
WAI score	79	24.25 (20.88-27)	24.00 (21.00-28.00)	0.00 (-2.00-3.00)	36	25.25 (21-31)	25.00 (21.25-29.38)	0.00 (-2.00-1.88)	-0.615	0.539	0.057
• Work ability	82	3 (0-5)	3 (0-6)	0 (-0.25-2)	36	3.5 (0.75-5.25)	2.5 (1-6)	0 (-1-1)	-1.164	0.244	-0.107
• Work	81	7.5	7	0	36	7	7	0	0.990	0.322	0.091

requirements	(6-9)	(6-8)	(-1-0.25)	(6-8.63)	(6-8.5)	(-1-1)

IQR = interquartile range.
